# Practices of Antifungal Prophylaxis Among Italian Pediatric Oncology–Hematology Centers

**DOI:** 10.1002/jha2.70337

**Published:** 2026-08-21

**Authors:** Martina Colli, Antonio Grasso, Manuela Spadea, Francesca Compagno, Lorenzo Chiusaroli, Paola Muggeo, Alessia Pancaldi, Rosamaria Mura, Daniela Onofrillo, Raffaella De Santis, Beatrice Mongelli, Angelica Barone, Simona Rinieri, Paola Coccia, Erica Ricci, Maura Faraci, Maria Rosaria D'Amico, Federica Galaverna, Pietro Gasperini, Letizia Brescia, Antonella Colombini, Riccardo Masetti, Francesco Baccelli, Simone Cesaro

**Affiliations:** ^1^ Pediatric Hematology and Oncology IRCCS Azienda Ospedaliero‐Universitaria di Bologna Bologna Italy; ^2^ Great Ormond Street Hospital for Children NHS Foundation Trust London UK; ^3^ Pediatric Onco‐Hematology, Stem Cell Transplantation and Cellular Therapy Division Regina Margherita Children's Hospital Turin Italy; ^4^ University of Turin Turin Italy; ^5^ Paediatric Haematology and Oncology Fondazione IRCCS Policlinico San Matteo Pavia Italy; ^6^ Division of Pediatric Infectious Diseases Department for Women's and Children's Health University Hospital of Padua Padova Italy; ^7^ Pediatric Oncology‐Hematology Unit Department of Pediatrics Azienda Ospedaliero Universitaria Policlinico Bari Italy; ^8^ Pediatric Oncology and Hematology Unit Department of Medical and Surgical Sciences for Mother, Children, and Adults University of Modena and Reggio Emilia Modena Italy; ^9^ Pediatric Onco‐Hematology Unit Azienda Ospedaliera Brotzu Cagliari Italy; ^10^ Hematology Unit Hospital of Pescara Pescara Italy; ^11^ Pediatric Hematology Oncology Unit Casa Sollievo della Sofferenza Hospital San Giovanni Rotondo Italy; ^12^ Pediatric Onco‐Hematology Unit Azienda Ospedaliero‐Universitaria di Parma Parma Italy; ^13^ Onco‐Hematology Day Hospital Pediatric Unit Sant'Anna Hospital Ferrara Italy; ^14^ Pediatric Onco‐Hematology Unit Azienda Ospedaliera delle Marche Ancona Italy; ^15^ Infectious Diseases Unit IRCCS Istituto Giannina Gaslini Genoa Italy; ^16^ Department of Pediatric Hemato‐Oncology AORN Santobono‐Pausilipon Naples Italy; ^17^ Department of Pediatric Hematology and Oncology and of Cell and Gene Therapy IRCCS Ospedale Pediatrico Bambino Gesù Rome Italy; ^18^ Department of Pediatric Hematology‐Oncology Azienda Unità Sanitaria Locale di Rimini Rimini Italy; ^19^ Pediatric Onco‐Hematology Unit Ospedale S.S. Annunziata Taranto Italy; ^20^ Department of Pediatrics Fondazione MBBM – Ospedale San Gerardo Monza Italy; ^21^ Department of Medical and Surgical Sciences (DIMEC) University of Bologna Bologna Italy; ^22^ University of Verona, Pediatric Hematology Oncology, Department of Mother and Child Azienda Ospedaliera Universitaria Integrata Verona Verona Italy

**Keywords:** antifungal prophylaxis, cancer, hematology, invasive fungal disease, oncology, pediatric

## Abstract

**Background:**

IFD is a significant cause of illness and death among pediatric patients undergoing chemotherapy or HSCT. Application of AFP in children varies widely and is frequently off‐label. This survey aims to describe real‐world AFP practices across centers within AIEOP and provide an estimate of IFD incidence.

**Methods:**

In the second half of 2023, AIEOP centers participated in a web‐based questionnaire collecting data on their AFP practices, antifungal agents, and IFD cases from 2021 to 2022.

**Results:**

Nineteen out of 44 AIEOP centers (43%) responded. AFP practices varied based on underlying disease and associated risk of IFD: it was used in 100% of centers treating AML, ALL‐Rel, and allo‐HSCT recipients, in 94.5% of centers for ALL‐HR patients, and in 53% of centers with ALL‐IR. Usage of AFP was limited (11%–35%) in low‐risk patients (ALL‐SR, HL, solid tumors). The most frequently used agents were liposomal amphotericin B and posaconazole for high‐risk patients, and fluconazole for lower‐risk patients. The estimated incidence of IFD was 4.7% (> 10% in ALL‐Rel, NHL, allo‐HSCT, SAA/AA).

**Conclusions:**

This survey shows variability in AFP drug choice. IFD rates remain elevated in high‐risk groups. These results underline the need for updated, pediatric‐specific national guidelines and antifungal stewardship to improve prophylaxis strategies.

**Trial Registration:**

The authors have confirmed clinical trial registration is not needed for this submission.

## 1 Introduction

IFD represents a relevant cause of morbidity and mortality among patients with cancer or those who have undergone HSCT. Molds and yeasts, such as *Candida* spp. and *Aspergillus* spp., are the most commonly implicated agents [1, 2, 3].

Patients present a high, low, or sporadic IFD risk based on their underlying diagnosis and neutropenic status. Those with AML, high‐risk ALL (ALL‐HR), relapsed ALL (ALL‐Rel), relapsed AML, SAA/AA, recipients of allo‐HSCT, and individuals with acute or chronic graft‐versus‐host disease (GVHD), along with persistent very severe neutropenia (ANC < 0.1 × 10^9^/L for over 14 days), are categorized as high risk [1, 4, 5, 6].

Moreover, knowledge of the local incidence and prevalent types of IFD is essential for defining the need for, and the type of, AFP [2, 4, 6, 7, 8, 9, 10, 13].

AFP is the recommended approach to decrease the occurrence of IFD. Pediatric guidelines advocate for primary AFP in patients with an incidence rate over 10% [4, 6, 9], indicating a high risk for IFD. Several studies have shown that pediatric patients with AML, ALL, NHL—whether in initial or relapse chemotherapy—as well as those undergoing HSCT, have a higher rate of IFD [11, 12]. Recently, the age ≥ 12 years and the poor early response to chemotherapy resulted in risk factors for pediatric ALL patients [5, 13]. Although the latest 2025 ECIL guidelines mainly focus on adults, they also include specific advice for pediatric patients, highlighting these factors as newly recognized risk indicators for IFD in children [5, 10, 11, 13].

While AFP represents a keystone of supportive care in high‐risk adult patients, its application in children remains less standardized, as indications are extrapolated from adult studies and may include the off‐label use of the newest antifungal drugs [14, 15, 16].

Children present unique challenges in antifungal management, including differences in pharmacokinetics, tolerability, and drug interactions [2, 16].

No published data on the use of AFP among pediatric centers in Italy are available. This survey aimed to describe AFP practices in Italian pediatric centers, assess adherence to existing guidelines, and provide a current estimate of IFD incidence.

## 2 Methods

This nationwide survey was conducted in the second semester of 2023 on the initiative of the Infectious Diseases Working Group of AIEOP. The first aim was to investigate the real‐world practices of AFP in pediatric patients undergoing chemotherapy or HSCT across Italian centers, including the antifungal drugs used and their indications. The secondary aim was to determine the number of IFDs that occurred at each center, stratified by underlying disease and AFP type used during the study period. The assessed period was 2021–2022, and we observed patients who underwent any treatment during that period. Centers reported the aggregate number of proven and probable IFD cases, defined by the 2019 EORTC/MSGERC criteria, encountered during the 2‐year survey period, the number of breakthrough IFD cases while on prophylaxis, and the total number of patients treated at each center for each disease subgroup [17].

An 8‐item web‐based questionnaire was sent to all 44 AIEOP centers (see Supporting Information). It examined the routine use of AFP across various conditions, including ALL, AML, MDS, SAA/AA, allo‐HSCT, auto‐HSCT, HL, B/T non‐Hodgkin lymphomas (NHL‐B, NHL‐T), and solid tumors such as rhabdomyosarcomas, Ewing's sarcoma, osteosarcomas, neuroblastomas, and central/peripheral nervous system tumors. For the ALL category, the risk levels—standard, intermediate, and high (e.g., ALL‐SR, ALL‐IR, ALL‐HR)—were defined according to criteria jointly established by AIEOP and the Berlin–Frankfurt–Munster (BFM) group [1]. Table 1 summarizes the risk classification of patients eligible for IFD, considering their underlying disease and remission status [1, 7, 8, 18].

**TABLE 1:** Definition of risk category among pediatric patients with cancer or after HSTC [1, 7, 8].

**High‐risk patients (> 10%)**	**Low‐risk patients (< 5%)**	**Sporadic cases**
High‐risk acute lymphoblastic leukemia (ALL‐HR)	Intermediate‐risk acute lymphoblastic leukemia (ALL‐IR)	Solid tumors
Relapsed/refractory ALL (ALL‐Rel)	Standard‐risk acute lymphoblastic leukemia (ALL‐SR)	Brain tumors
Acute myeloid leukemia (AML)	Non‐Hodgkin lymphoma (NHL)	Hodgkin' s lymphoma (HL)
Allogeneic hematopoietic stem cell transplantation (allo‐HSCT)		
Grade 2–4 acute graft‐versus‐host disease (GVHD)/chronic GVHD		

For each disease, respondents indicated the type of AFP agents from a predefined list: fluconazole (F), voriconazole (V), posaconazole (P), amphotericin B (A), echinocandins (E), isavuconazole (I), or no prophylaxis (N). Data collection and processing complied with Italian laws on patient confidentiality and good clinical practice. Data were summarized as counts and percentages. Continuous variables were reported as medians with interquartile ranges. The incidence of IFD was estimated from aggregated, center‐level counts of underlying diagnoses and documented IFD events.

In Table 2, we summarized the key recommendations for AFP.

**TABLE 2:** Summary of prophylaxis recommendations [8, 9, 23, 32].

Recommending body	Posaconazole	Fluconazole	Itraconazole	Liposomal amphotericin B	Caspofungine	Voriconazole	Isavuconazole
8th ECIL Guidelines–2020 [12]	Spectrum: molds + yeasts 1 m–12 y: oral suspension > 13 y: delayed release tablet TDM: ≥ 0.7 mg/l First‐line agent in high‐risk patients	Spectrum: yeasts EV/OS Recommended for patients with leukemia and allo‐HSC (pre‐engraftment phase)	Spectrum: molds + yeasts > 18 y TDM: ≥ 0.5 mg/l	Spectrum: molds + yeasts EV (daily or twice per week) Alternative option in case of no tolerance/contraindication to triazoles (not approved for prophylaxis)	Spectrum: molds Ev (once day)	Spectrum: molds + yeasts Ev/OS > 2y TDM: ≥ 1–5 mg/L Alternative agent for IFD prophylaxis due to higher rates of adverse events (liver function abnormalities) and variable metabolism.	Spectrum: aspergillosis and mucormicosis Ev Os
Grading	GR: A	GR: A	GR: B	GR: B	GR: none	GR: B	Recent case series/revies in the pediatric patients [9, 26].
10th ECIL Guidelines–2025	Spectrum: molds + yeasts. Approved for ≥ 2 y.o. (IV, PFS); delayed‐release tabs > 40 kg. First‐line in AML, MDS, HSCT.						
	Spectrum: yeasts. Used for mucosal prophylaxis (e.g., auto‐HSCT, low mold risk allo‐HSCT).	Spectrum: molds + yeasts. SUBA form better tolerated. Classic itraconazole downgraded (C‐I).	Spectrum: molds + yeasts. Not approved for prophylaxis. Consider only in intolerance to azoles.	Spectrum: molds. Pediatric & adult AML/MDS: B‐I/B‐II. Also allo‐HSCT.	Spectrum: molds + yeasts. Effective but high risk of AEs. Not first‐line.	Spectrum: aspergillosis + mucormycosis. Alternative if QTc or liver concerns. Approved for pediatrics.	
Grading	A‐I (AML/MDS), A‐I (HSCT)	B‐I to B‐III depending on context	C‐I (classic), C‐II (SUBA)	C‐II	B‐I (pediatrics), B‐II (adults)	B‐II	B‐II
Notes	First‐line in AML induction, MDS, allo‐HSCT; also approved in pediatrics > 2 y.o.	Used when mold risk is low or only for mucosal candidiasis	Not preferred over posaconazole due to inconsistent PK	Toxicity limits use; only for intolerance or resistance	Upgraded based on new evidence in AML, allo‐HSCT	Use with caution for IFD prophylaxis due to liver and PK variability	Proposed alternative in AML, allo‐HSCT, especially with QTc issues

## 3 Results

Nineteen of 44 AIEOP centers completed the questionnaire, yielding a response rate of 43% (19/44). All these centers served as regional referral centers for the diagnosis of malignant hematological diseases, and 12 of them also performed allo‐HSCT. Their geographic distribution spans nearly the entire peninsula, making them a broadly representative sample.

### 3.1 AFP Practices

Table 3 summarizes the use of AFP versus no AFP by underlying disease and type of AFP used. AFP was adopted by 100% of centers in high‐risk conditions, specifically AML (both first diagnosis and relapse), ALL‐Rel, and allo‐HSCT recipients, while it was used in 94.5% of centers in ALL‐HR and in 53% of centers in ALL‐IR. In contrast, only 11.1%–35.3% of centers adopted AFP in patients with ALL‐SR, HL, and solid tumors. Variable rates of AFP were reported in other settings: 86.6% in auto‐HSCT, 60% in NHL‐T‐Rel, 57.1% in NHL‐B‐Rel, 73.3% in SAA, and 50% in MDS.

**TABLE 3:** Summary of antifungal prophylaxis used in each disease category among centers. The type of prophylaxis reported by centers included in some disease category more options, for example for HSCT patients.

**Group**	**Centers**	**Prophylaxis no**	**Prophylaxis yes**	**Fluconazole**	**Voriconazole**	**Posaconazole**	**L‐AmB**	**Echinocandine**	**Isavuconazole**	**Itraconazole**
ALL‐SR	17/19	11 (64.7%)	6 (35.3%)	3	0	0	3	0	0	0
ALL‐IR	17/19	8 (47%)	9 (53%)	5	0	1	4	0	0	0
ALL‐HR	18/19	1 (5.5%)	17 (94.5%)	4	2	7	10	0	0	0
ALL‐Rel	17/19		17 (100%)	3	2	7	13	0	0	0
AML	17/19		17 (100%)	1	2	8	13	0	0	0
AML‐Rel	15/19		15 (100%)	0	2	7	12	0	0	0
Allo‐HSCT	12/19		12 (100%)	5	3	6	8	2	1	0
Auto‐HSCT	15/19	2 (13.4%)	13 (86.6%)	10	1	3	3	0	0	0
NHL‐B	17/19	12 (70.5%)	5 (29.5%)	4	1	1	1	0	0	0
NHL‐T	17/19	11 (64.7%)	6 (35.3%)	3	2	2	3	0	0	0
NHL‐B Rel	14/19	6 (42.9%)	8 (57.1%)	2	2	3	4	0	0	0
NHL‐T Rel	15/17	6 (40%)	9 (60%)	2	2	4	5	0	0	0
HL	18/19	16 (88.9%)	2 (11.1%)	2	0	0	0	0	0	0
HL‐Rel	17/19	13 (76.5%)	4 (23.5%)	1	1	2	2	0	0	0
MDS	16/19	8 (50%)	8 (50%)	1	2	5	2	1	0	1
SAA/AA	15/19	4 (26.7%)	11 (73.3%)	2	2	5	7	1	0	1
Solid tumor	18/19	16 (88.9%)	2 (11.1%)	2	0	0	0	0	0	0

Abbreviation: ALL‐HR, acute lymphoblastic leukemia–high risk; ALL‐IR, acute lymphoblastic leukemia–intermediate risk; Allo‐HSCT, allogeneic hematopoietic stem cell transplantation; ALL‐Rel, relapsed acute lymphoblastic leukemia; ALL‐SR, acute lymphoblastic leukemia–standard risk; AML, acute myeloid leukemia; AML‐Rel, relapsed acute myeloid leukemia; Auto‐HSCT, autologous hematopoietic stem cell transplantation; HL, Hodgkin lymphoma; HL‐B, B‐cell non‐Hodgkin lymphoma; HL‐Rel, relapsed Hodgkin lymphoma; HL‐T, T‐cell non‐Hodgkin lymphoma; L‐AMB, amphotericin B; MDS, myelodysplastic syndrome; NHL‐B‐Rel, relapsed B‐cell non‐Hodgkin lymphoma; NHL‐T‐Rel, relapsed T‐cell non‐Hodgkin lymphoma; SAA/AA, severe aplastic anemia/aplastic anemia.

As shown in Table 4, the most frequently adopted antifungal agents across all categories were liposomal amphotericin B and posaconazole, particularly in high‐risk settings such as AML, ALL‐HR, relapsed diseases, and HSCT. Fluconazole was frequently used in both lower‐ and high‐risk settings. Voriconazole was used more selectively, mostly in relapsed cases. Echinocandins and itraconazole were prescribed infrequently and limited to specific cases (e.g., MDS, SAA/AA).

**TABLE 4:** Policy of antifungal prophylaxis used by centers according to the type of underlying disease and the remission status or the phase of treatment (more than one option was possible for each disease category).

**Disease**	**Fluconazole**	**Posaconazole**	**Voriconazole**	**Echinocandine**	**Isavuconazole**	**Itraconazole**	**Liposomal amphotericin B**
ALL‐SR	73%	—	—	—	—	—	27%
ALL‐IR	65.5%	10%	—	—	7%	—	34.5%
ALL‐HR	18%	14%	13%	—	—	—	81%
ALL‐Rel	8%	16%	11%	—	—	—	84%
AML	1%	21%	10%	—	—	—	77%
AML‐Rel	—	30%	3%	—	—	—	86.5%
Allo‐HSCT	73%	36.5%	13%	8%	5%	—	87%
Auto‐SCT	99%	4%	—	—	—	—	54%
HL	100%	—	—	—	—	—	—
HL‐Rel	83%	8%	8%	—	—	—	17%
MDS	—	79%	26%	2%	—	14%	22%
NHL‐B	91.5%	5%	5%	—	—	—	—
NHL‐B‐Rel	18%	—	—	—	—	—	82%
NHL‐T	100%	—	—	—	—	—	—
NHL‐T‐Rel	—	—	—	—	—	—	100%
SAA/AA	11.5%	61.5%	15%	4%	—	8%	58%
Solid tumor	100%	—	—	—	—	—	—

Abbreviation: ALL‐HR, acute lymphoblastic leukemia–high risk; ALL‐IR, acute lymphoblastic leukemia–intermediate risk; Allo‐HSCT, allogeneic hematopoietic stem cell transplantation; ALL‐Rel, relapsed acute lymphoblastic leukemia; ALL‐SR, acute lymphoblastic leukemia–standard risk; AML, acute myeloid leukemia; AML‐Rel, relapsed acute myeloid leukemia; Auto‐HSCT, autologous hematopoietic stem cell transplantation; HL, Hodgkin lymphoma; HL‐B, B‐cell non‐Hodgkin lymphoma; HL‐Rel, relapsed Hodgkin lymphoma; HL‐T, T‐cell non‐Hodgkin lymphoma; MDS, myelodysplastic syndrome; NHL‐B‐Rel, relapsed B‐cell non‐Hodgkin lymphoma; NHL‐T‐Rel, relapsed T‐cell non‐Hodgkin lymphoma; SAA/AA: severe aplastic anemia/aplastic anemia.

### 3.2 Proportion of Patients With IFD

The crude incidence over the study period was 4.7% (129 cases out of 2697), irrespective of antifungal prophylaxis use (Table 5). The crude incidence of IFD was: 0.5% (3/514) for solid tumors, 0.7% (2/263) for auto‐HSCT, 4.1% (7/168) for ALL‐HR, 5.5% (13/238) for ALL‐IR, 6.2% (1/4) for NHL‐T‐Rel, 7.7% (42/542) for allo‐HSCT, 8.1% (12/148) for AML, 9.5% (16/168) for ALL‐SR, 10% (3/31) for NHL‐T, 10.7% (3/28) for SAA, 12.3% (16/130) for ALL‐Rel, 13.5% (5/37) for AML‐Rel, and 1.6% (1/62) for MDS.

**TABLE 5:** Number of Probable/Proven IFD according to the disease category and prophylaxis administration among all centers.

**Underlying disease**	***N* of patients (%)**	**Patients on antifungal prophylaxis (%)**	***N* of proven‐probable IFD (%)**
ALL‐SR	168 (6.2%)	74 (44%)	16 (9.5%)
ALL‐IR	238 (8.8%)	113 (47.5%)	13 (5.5%)
ALL‐HR	168 (6.2%)	153 (91%)	7 (4.1%)
ALL‐Rel	130 (4,8%)	120 (92%)	16 (12.3%)
AML	148 (5.5%)	145 (98%)	12 (8.1%)
AML‐Rel	37 (1.3%)	37 (100%)	5 (13.5%)
Allo‐HSCT	542 (20%)	542 (100%)	42 (7.7%)
Auto‐HSCT	263 (9.7%)	214 (81%)	2 (0.7%)
NHL‐B	97 (3.6%)	48 (49%)	2 (2%)
NHL‐T	31 (1%)	12 (39%)	3 (10%)
NHL‐B‐Rel	16 (0,6%)	13 (93.5%)	3 (18.7%)
NHL‐T‐Rel	4 (0.1%)	2 (50%)	1 (6.2%)
HL	226 (8.4%)	59 (61%)	0
HL‐Rel	25 (0.9%)	10 (40%)	0
MDS	62 (2.3%)	2 (3%)	1 (1.6%)
SAA/AA	28 (1%)	26 (93%)	3 (10.7%)
Solid tumors	514 (20%)	40 (8%)	3 (0.5%)
Total	2697	1664 (62%)	129 (4.7%)

Abbreviation: ALL‐HR, acute lymphoblastic leukemia–high risk; ALL‐IR, acute lymphoblastic leukemia–intermediate risk; Allo‐HSCT, allogeneic hematopoietic stem cell transplantation; ALL‐Rel, relapsed acute lymphoblastic leukemia; ALL‐SR, acute lymphoblastic leukemia–standard risk; AML, acute myeloid leukemia; AML‐Rel, relapsed acute myeloid leukemia; Auto‐HSCT, autologous hematopoietic stem cell transplantation; HL, Hodgkin lymphoma; HL‐B, B‐cell non‐Hodgkin lymphoma; HL‐Rel, relapsed Hodgkin lymphoma; HL‐T, T‐cell non‐Hodgkin lymphoma; MDS, myelodysplastic syndrome; NHL‐B‐Rel, relapsed B‐cell non‐Hodgkin lymphoma; NHL‐T‐Rel, relapsed T‐cell non‐Hodgkin lymphoma; SAA/AA, severe aplastic anemia/aplastic anemia).Clinical Trial Registration Information ClinicalTrials.gov ID: NCT06148675 (https://clinicaltrials.gov/study/NCT06148675.

No proven‐probable IFD was reported for HL and Rel‐HL patients. Notably, in ALL‐SR, IFD was reported only in patients not using AFP. In ALL patients, 7 of 13 IFD cases were in patients receiving AFP (fluconazole, posaconazole, and liposomal amphotericin B). In ALL‐HR, all IFD cases except one (15/16) were in patients on AFP (fluconazole, posaconazole, voriconazole, and liposomal amphotericin B). All 42 IFD cases reported in patients with AML, AML‐Rel, NHL‐T, NHL‐B‐T‐Rel, MDS, and SAA occurred in patients on AFP. In auto‐HSCT patients, only two IFD cases were reported, and one involved AFP.

## 4 Discussion

Although there is general agreement on using AFP in high‐risk patients for IFD, its application in pediatric hematology‐oncology remains debated. The main challenges are the limited pediatric data on efficacy and safety from randomized studies, as well as the lack of prescribability of some recent antifungal drugs used for prophylaxis in adults. In Table 2, we summarize the key recommendations for AFP, highlighting similarities and differences regarding indications, preferred agents, and clinical strategies. In the pediatric population, guidelines recommend risk‐based approaches and endorse posaconazole as the first‐line agent for high‐risk patients, with fluconazole for low‐risk cases. Alternatives include itraconazole, voriconazole, liposomal amphotericin B, micafungin, and caspofungin. Although not approved, isavuconazole is a potential salvage option [5, 8, 13, 21,–24]. The latest ECIL 2025 guidelines significantly strengthen and clarify these recommendations, incorporating new evidence and more accurately stratifying risk. They emphasize that children with ALL aged ≥ 12 years and/or showing poor response to induction therapy are classified as high‐risk for IFD, requiring prophylaxis [4, 19, 20]. In Italy, approved options for prophylaxis include posaconazole, itraconazole, and micafungin in selected high‐risk settings, mainly acute myeloid leukemia, with the addition of fluconazole and voriconazole in patients undergoing allogeneic HSCT. Therefore, several prophylactic strategies reported in this survey also reflect an off‐label of antifungal prophylaxis policy adopted by some centers and based on individual patient risk assessment, local epidemiology, drug availability, local hospital cost authorization, and multidisciplinary clinical judgement (antifungal stewardship).

Our study revealed that 62% of patients received AFP, with significant variation across centers influenced by disease type, treatment intensity, and perceived IFD risk. The use of AFP was almost universal in high‐risk groups such as AML, ALL‐Rel, and allo‐HSCT, with adoption rates ranging from 94.5% to 100%. These figures align with international guidelines and aim to reduce morbidity and mortality from IFD. Interestingly, a sizable portion of patients at intermediate or standard risk still received AFP. In ALL‐IR, 53% of centers used AFP, while in ALL‐SR, HL, and solid tumors, usage ranged from 11.1% to 35%. We attribute this variation to differences in institutional practices, local epidemiology, or clinicians' personal judgment.

Despite prophylaxis, ALL‐IR and ALL‐SR patients exhibited IFD incidence of 5.5% and 9.5%, respectively. These figures highlight inconsistent use of prophylaxis among groups not traditionally considered high risk and emphasize the need to reclassify these patients based on both conventional risk assessments and additional factors, as recommended by the latest ECIL guidelines. It also points to the importance of prospective research to identify more personalized risk factors.

Fluconazole, primarily effective against yeasts, is frequently used for low‐ to intermediate‐risk IFD, including ALL‐SR, ALL‐IR, HL, NHL, and auto‐HSCT. It remains recommended in low‐risk situations, such as for leukemia patients or those undergoing allo‐HSCT during pre‐engraftment, particularly when mold infection incidence is low or early mold‐detection algorithms are used [10, 25]. The ECIL 2025 guidelines advise careful use of fluconazole, stressing the value of combining it with mold‐specific surveillance when necessary [9, 10, 25].

Liposomal amphotericin B is frequently used for AFP, especially in ALL patients and those at high risk of IFD. Although it is not officially approved for AFP, the possibility to administer it intermittently two to three times a week and its minimal drug interactions with chemotherapy make it a useful alternative as a mold‐active AFP, particularly when there is no indication for triazoles because of drug interactions, side effects, or gastrointestinal malabsorption [4, 10, 25, 26].

Posaconazole is the most commonly used triazole by AIEOP centers, particularly for high‐risk patients such as those with AML, ALL‐Rel, and allo‐HSCT. It is also the recommended first‐line treatment for AFP in high‐risk patients due to its broad activity against both yeasts and molds.

Posaconazole is approved as delayed‐release tablets for children aged 12 years and older, and as oral suspension or intravenous formulations for those aged 2 years and older weighing more than 40 kg, based on recent pediatric studies that confirm safety and efficacy. Therapeutic drug monitoring (TDM) is recommended, aiming for a target trough level of at least 0.7 mg/L [4, 8, 9, 10].

Voriconazole and echinocandins were rarely used, whereas isavuconazole was used at only a single center in the allogeneic HSCT group.

Voriconazole, approved for children aged 2 and above, has broad‐spectrum antifungal activity against *Aspergillus* spp. Nonetheless, its application in pediatric prophylaxis is constrained due to notable liver toxicity and variability in how patients metabolize the drug. Monitoring serum levels through TDM is advised to keep concentrations between 1 and 5 mg/L [4, 10].

The survey indicated limited use of old and newer or more targeted antifungals such as echinocandins, itraconazole, and isavuconazole. This finding likely stems from concerns about itraconazole's tolerability and pharmacokinetics, as well as the limited availability and clinical experience with isavuconazole in pediatric populations. Itraconazole is not approved for individuals under 18, and pediatric data are scarce. The newer SUBA‐itraconazole formulation, which offers improved bioavailability, might be a future option, but it still lacks comprehensive pediatric data, and it is not approved by the European Medicines Agency. TDM remains recommended for itraconazole, aiming for a level of ≥ 0.5 mg/L [4]. Other novel antifungal agents and formulations are currently under clinical development and may contribute to future prophylactic strategies. Among these, rezafungin, a long‐acting echinocandin designed for once‐weekly administration, and inhaled opelconazole, a triazole formulation aimed at achieving high pulmonary exposure while limiting systemic drug–drug interactions, are of potential interest. However, their role in pediatric hematology‐oncology remains undefined, and no current pediatric prophylaxis guideline supports their routine use [24, 25, 26].

Finally, isavuconazole, a broad‐spectrum triazole effective against *Aspergillus* spp. and Mucorales, is not approved for primary prophylaxis, particularly in children. However, recent ECIL‐10 guidelines recommend it as a second‐line agent for patients intolerant to posaconazole or voriconazole, especially in cases of QT prolongation or hepatotoxicity. Although randomized clinical data in children are lacking, case series suggest it could be a salvage or tailored alternative in select pediatric cases with prior azole exposure [4, 10, 25].

A significant drawback of all triazoles is their interaction with vincristine, a crucial drug for induction and reinduction chemotherapy in ALL patients, which prevents the safe use of this antifungal class in these patients. Interestingly, isavuconazole has a lower risk of interacting with vincristine, and its tolerability and safety in ALL patients warrant further clinical research [27, 28].

Micafungin, an intravenous echinocandin, is approved for the prevention of invasive *Candida* infections in neutropenic patients. Despite the narrow spectrum, it is recommended for specific situations, such as in patients with AML or those undergoing allogeneic HSCT, especially when triazoles are not an option [4].

Caspofungin, an echinocandin, protects against *Candida* species and certain molds, such as *Aspergillus* species. Recent prospective trials have shown its effectiveness in preventing invasive fungal diseases, especially in patients with ALL aged 12 years or older or those with high‐risk factors [9].

The rough estimate of proven‐probable IFD from the survey indicates that the incidence of IFD remains significant, especially in high‐risk categories such as ALL‐Rel, NHL, AML, SAA/AA, and allo‐HSCT. These findings are consistent with the literature, which reports a mean incidence of around 4.7% across different centers in high‐risk groups. The incidence of IFD ranged from 5% to 10% in the ALL‐SR and ALL‐IR categories, where AFP is not strictly recommended [10]. The lowest IFD incidence (< 1%) was observed in patients with solid tumors, HL, and those undergoing auto‐HSCT. Conversely, the highest rates (> 10%) were observed in patients with ALL‐Rel and AML‐Rel (12.3%–13.5%), SAA/AA (10.7%), and NHL–B‐Rel (18.7%) [8].

We must underline that breakthrough IFD was reported among patients receiving prophylaxis; however, because data were collected at an aggregated center level, it was not possible to reliably determine the denominator of patients exposed to each individual prophylactic agent or to compare breakthrough rates between mold‐active and yeast‐active prophylaxis. Therefore, this survey's limitation is that the data do not allow for the correlation between IFD occurrence and AFP use. Gathering this vital information would have required a study reporting individual patient data rather than aggregate data, as was the case here. Clinically, this information is essential because it helps decide intervention strategies, such as increasing primary AFP use or focusing on preventing or treating breakthrough IFD. Causes of breakthrough IFD can include using inadequate antifungal agents (e.g., non‐mold‐active AFP), poor bioavailability of oral azole AFP in patients with severe mucositis or malabsorption, and drug–drug interactions that prevent reaching therapeutic antifungal levels.

Interestingly, the survey revealed that specific patient categories, such as HL, HL‐Rel, solid tumors, and auto‐HSCT, have an IFD occurrence of less than 1% or none at all. For solid tumors, AFP use was minimal and likely limited to patients with advanced sarcoma, neuroblastoma, or HL‐Rel who require intensive chemotherapy. The high frequency of AFP use in auto‐HSCT appears unwarranted, given the low incidence of IFD. Utilizing peripheral stem cells for auto‐HSCT facilitates rapid myeloid recovery, significantly shortening the period of severe neutropenia, the leading risk factor for IFD in these patients.

However, considering that several disease subgroups included very small denominators, particularly SAA/AA, relapsed NHL, and relapsed HL, the corresponding IFD proportions should be interpreted cautiously and considered descriptive only. Moreover, patients with solid tumors represented a large subgroup of the observed population, but their IFD incidence was very low, suggesting excluding this group in the future from the computation of the incidence of IFD in the pediatric hematology setting.

The overall IFD incidence of 4.7% observed in this survey should not be considered sufficient to support routine antifungal prophylaxis in all pediatric oncology‐hematology patients. Rather, given the heterogeneity of the population, an individualized approach is crucial for guiding appropriate AFP in children, especially given the growing use of new therapies such as immunotherapy and cellular therapy in pediatric care [24]. Recent studies emphasize that, while antifungal prophylaxis is necessary in pediatric settings, it must be tailored to patient characteristics and risk factors [29, 30]. In this context, multidisciplinary discussions, combined with robust antimicrobial stewardship, are vital to ensure that effective prophylactic strategies are in place [22, 29, 30, 31, 32]

## 5 Conclusions

To our knowledge, this is the first study to analyze real‐world use of antifungal prophylaxis in Italian pediatric hematology–oncology centers. The findings showed significant variation in both the choice of antifungal agents and the underlying diseases prompting prophylaxis, reflecting the lack of unified national guidelines and differences in local risk assessment methods. Because of its retrospective, descriptive nature, this survey could not establish a causal link between antifungal prophylaxis use and IFD occurrence, or evaluate how different prophylactic strategies influence the type and outcome of IFD. This survey highlights the necessity for prospective studies with comprehensive data collection to clarify AFP's effectiveness across various patient groups. Despite this, by illustrating current practices across multiple Italian centers, the study provides valuable insights into prevailing trends and disparities, laying the groundwork for the development of shared, evidence‐based national guidelines for antifungal prophylaxis in children with hematological and oncological malignancies.

## Funding

This study was supported by the University of Verona.

## Consent

This study was based on a retrospective, non‐interventional survey collecting anonymized, aggregated data at the center level from participating AIEOP pediatric hematology–oncology centers. No directly identifiable patient information was collected. Data handling complied with Italian laws on data protection and good clinical practice.

## Conflicts of Interest

The authors declare no conflicts of interest

## Supporting information




**Supporting Information**: jha270337‐sup‐0001‐SuppMat.docx

## Data Availability

Data are available upon reasonable request.
