# Supplementary material for: Practices of Antifungal Prophylaxis Among Italian Pediatric Oncology–Hematology Centers
Source: EJHaem. 2026 Aug 21;7(4):e70337. doi: 10.1002/jha2.70337 (PMC13495061; doi:10.1002/jha2.70337)
Supplement: Supplementary file 1 [file JHA2-7-e70337-s001.docx]

**Supplementary material**

**Table 1. Survey form**

| Disease | No | yes | Type of AF prophylaxis | Number of diagnoses 2021 | Number of diagnoses 2022 |
| --- | --- | --- | --- | --- | --- |
| ALL-SR |  |  |  |  |  |
| ALL-IR |  |  |  |  |  |
| ALL-HR |  |  |  |  |  |
| ALL-relapsed |  |  |  |  |  |
| AML |  |  |  |  |  |
| AML-relapsed |  |  |  |  |  |
| Allo-HSCT |  |  |  |  |  |
| Auto-HSCT |  |  |  |  |  |
| NHL-B |  |  |  |  |  |
| NHL-T |  |  |  |  |  |
| NHL-B relapsed |  |  |  |  |  |
| NHL-T relapsed |  |  |  |  |  |
| LH |  |  |  |  |  |
| LH relapsed |  |  |  |  |  |
| MDS |  |  |  |  |  |
| SAA/AA |  |  |  |  |  |
| Solid Tumor  specify ewing, osteosarcoma, wilms |  |  |  |  |  |
